# Suturing a Wounded Heart

**Published:** 1903-01-10

**Authors:** 


### SUTURING A WOUNDED HEART.

A case is reported by Dr. L. L. Hill,[1] of Alabama, in which a wound of the heart was successfully treated by suture. The patient was a negro, 13 years of age, who had been stabbed. The knife blade entered the fifth intercostal space about a quarter of an inch to the right of the left nipple, and penetrating the apex of the heart passed into the left ventricle. The wound was about three-eighths of an inch long. On seeing the patient Dr. Hill found that the radial pulse was almost imperceptible and the heart sounds were heard with difficulty. He had dyspnœa and was very restless. His extremities were cold as also were his lips and nose, and his countenance showed great distress. Eight hours after the infliction of the wound the operation was undertaken. A flap containing 4 inches of third, fourth and fifth ribs was turned up towards the sternum, the cartilages acting as the hinge. There was no blood in the pleural cavity, but the pericardium was enormously distended. On incising this about ten ounces of blood were evacuated. The pulse immediately improved. The heart was then lifted up and steadied while a catgut suture was passed through the centre of the wound in the ventricle and the hæmorrhage was thus controlled. The cavities of both pericardium and pleura were cleansed with saline solution, the pericardium was then closed with seven catgut sutures and the pleural cavity drained with iodoform gauze. The musculo-osseous flap was then brought down and stitched in position. There was delirium and some rise of temperature, but he sat up on the fifteenth day, and got well.

[1] New York Medical Record, Nov. 29.

